# 2603. Acceptance of Pharmacist-Led Stewardship Recommendations for Patients with Community-Acquired Pneumonia

**DOI:** 10.1093/ofid/ofad500.2217

**Published:** 2023-11-27

**Authors:** Ramara E Walker, Andrea Pallotta, Rebecca Schulte, Larisa Tereshchenko, Victoria A Criswell, Abhishek Deshpande, Michael Rothberg

**Affiliations:** Cleveland Clinic, Cleveland, Ohio; Cleveland Clinic, Cleveland, Ohio; Cleveland Clinic, Cleveland, Ohio; Cleveland Clinic, Lerner Research Institute, Cleveland, Ohio; Cleveland Clinic, Cleveland, Ohio; Cleveland Clinic, Cleveland, Ohio; Cleveland Clinic, Cleveland, Ohio

## Abstract

**Background:**

Community-acquired pneumonia (CAP) is a leading cause of hospitalizations and mortality in the US. Overuse of extended spectrum antibiotics (ESA) for CAP contributes to antimicrobial resistance. The 2019 Infectious Diseases Society of America/American Thoracic Society guidelines emphasize de-escalation of ESA following negative cultures, early switch to oral (PO) antibiotics and limited duration of therapy (DOT). This study describes physician acceptance of pharmacist-led stewardship recommendations in hospitalized CAP patients.

**Methods:**

This intervention is part of an ongoing, large, pragmatic, 2×2 factorial, cluster-randomized, controlled trial across 12 Cleveland Clinic hospitals evaluating the independent and combined effects of pharmacist-led de-escalation and rapid diagnostic testing versus usual care in adults with CAP. The pharmacist developed and delivered the following interventions: ESA de-escalation, DOT, intravenous (IV) to PO transition, and antimicrobial discontinuation. Descriptive statistics were used to describe acceptance rates by prescribers for each pharmacist intervention types.

**Results:**

From November 1, 2022 to March 16, 2023, the pharmacist recommended 317 interventions for 212/1533 CAP patients (14%). Of the recommended interventions, 57.5% were for patients receiving an ESA during their hospitalization, and 16% were for patients classified as having severe CAP. ESA de-escalation, DOT, IV to PO transition, antimicrobial discontinuation, and other interventions made up 22%, 45%, 18%, 11%, and 4% of the total recommended interventions, respectively. Providers accepted 238 recommendations (75%); they were most likely to accept recommendations for ESA de-escalation (60/69, 87%) and IV to PO transition (48/56, 86%), followed by antimicrobial discontinuation (26/35, 74%), and other (10/13, 77%). Recommendations to limit DOT were least likely to be accepted (94/144, 65%). CAP recommendations were made to the following prescribers: physicians (83%) and advanced practice providers (17%) with equal acceptance rate (75%).

**Figure:**
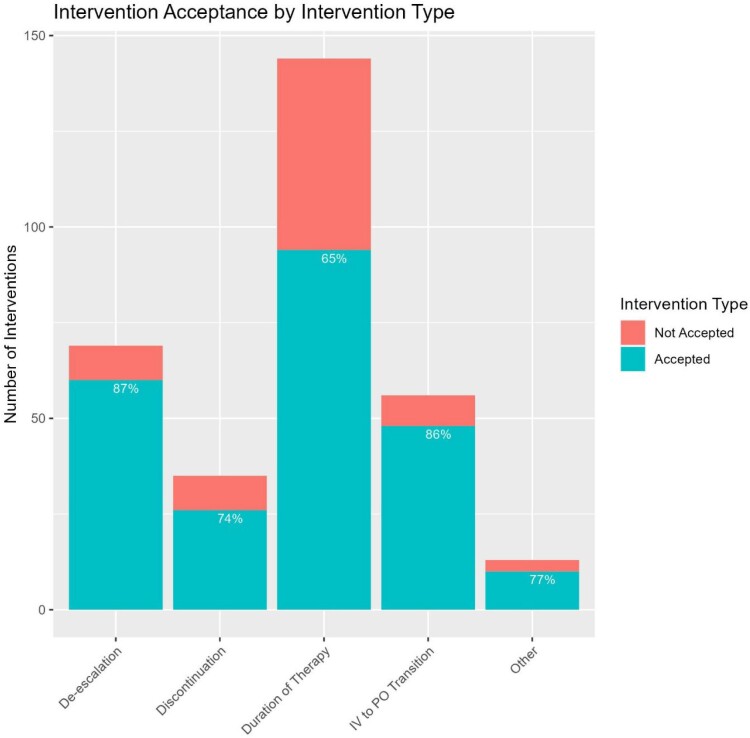

Summary of CAP-targeted intervention acceptance rates by intervention category

**Conclusion:**

Approximately 1 in 7 patients garnered a CAP recommendation. While most recommendations were accepted, prescribers were most receptive to de-escalating ESA and least receptive to limiting DOT.

**Disclosures:**

**Abhishek Deshpande, MD; PhD**, Merck: Advisor/Consultant|Seres Therapeutics: Grant/Research Support **Michael Rothberg, MD, MPH**, Blue Cross Blue Shield: Advisor/Consultant

